# Can bioelectrical impedance analysis be used to identify water loading in patients with anorexia nervosa?– Implications from experimental measurements in young, healthy, and normal weight women

**DOI:** 10.1186/s40337-025-01285-z

**Published:** 2025-05-19

**Authors:** Nadja Knoll-Pientka, Dorina Schils, Katrin Pasternak, Sandra Czarnetzky, Christoph Jansen, Gertraud Gradl-Dietsch, Jochen Seitz, Eva-Maria Skoda, Lars Libuda, Adam Schweda, Martin Teufel

**Affiliations:** 1 Clinic for Psychosomatic Medicine and Psychotherapy, University of Duisburg-Essen, LVR-University Hospital Essen, Essen, Germany; 2 Center for Translational Neuro- and Behavioral Sciences (C-TNBS), University of Duisburg-Essen, Essen, Germany; 3 Department of Child and Adolescent Psychiatry, Psychosomatics and Psychotherapy, University of Duisburg-Essen, LVR-University Hospital Essen, Essen, Germany; 4 Faculty of Natural Sciences, Institute of Nutrition, Consumption and Health, Paderborn University, Paderborn, Germany

## Abstract

Anorexia nervosa (AN) is a severe mental disorder, and patients with AN are characterized by a low body weight and a fear of gaining weight. Restoration of body weight to the normal range is one major treatment aim, which can be a challenging process for the patients. Hence, as a psychopathological symptom of AN, weight manipulations such as water loading before weighing are commonly observed in clinical routine. Biological impedance analysis (BIA) is a helpful tool in routinely visualizing changes in body composition during the refeeding process. Here, we targeted the question whether BIA could potentially detect water loading in healthy, young and normal weight women serving as a preclinical model for patients with AN.

Sixty-one women (mean ± SD, 22.2 ± 2.2 years, 20.4 ± 1.0 kg/m^2^) were included in the analyses. We used a full experimental setting with a cross-over design on two consecutive days. On both days, all participants underwent a baseline BIA in the fasting state (t_0_). Directly thereafter, participants either consumed 1000 ml of tap water (intervention-condition) or waited for the second BIA measurement 20 min (t_1_) after baseline (control-condition), and vice versa the subsequent day. Two further BIA measurements took place at 40 (t_2_) and 60 min (t_3_) after baseline.

After water consumption, we found increases in derived fat mass (FM) and phase angle at t_1_ to t_3_, decreases at t_1_ and subsequent increases at t_2_ and t_3_ in extracellular water (ECW) and total body water (TBW). In contrast, skeletal muscle mass (SMM) and ECW/TBW remained rather stable. In the control-condition, most parameters remained constant.

Our study provided insight into the changes of impedance raw data and derived body compartments after water consumption among young, healthy and normal weight women. Although the considerable increase of FM in combination with a rather stable course of SMM, as found in our experiment after the consumption of water, could be a potential hint for water loading, further investigation considering the limitations of the present study as for instance the different metabolisms of patients with AN vs. healthy women is required, before transferability to the clinical setting will be given.

**Supplementary Information:**

The online version contains supplementary material available at 10.1186/s40337-025-01285-z.

## Abstract (plain language summary)

Anorexia nervosa (AN) is a severe mental disorder. Patients with AN are characterized by a low body weight and a fear of gaining weight. The restoration of a body weight into the normal range is one major aim in the treatment of these patients. Weight manipulations such as drinking before being weighted are part of the disease and commonly observed in clinical practice among patients with AN. Bioelectrical impedance analysis (BIA) is an easy-to-use method to determine body composition and may be supportive in the treatment process. Therefore, we performed an experiment with young, healthy, and normal weight women, to see whether the consumption of 1000 ml of tap water can be detected by BIA. We observed an increase in several body compartments such as fat mass, while other parameters such as skeletal muscle mass remained stable. Overall, our results might provide a good basis to make drinking “visible” using BIA. Nevertheless, further investigation regarding the differences between healthy women and patients with AN will be necessary, before BIA can be used in the clinical setting for the detection of drinking before being weighted among patients with AN.

**Supplementary Information:**

The online version contains supplementary material available at 10.1186/s40337-025-01285-z.

## Introduction

Anorexia nervosa (AN) is a severe mental disorder associated with an increased mortality risk [1]. The fear of gaining weight in combination with a very low body weight are key features of AN [2] with potentially detrimental consequences for a variety of organ systems, such as the cardiovascular or endocrine system [3]. Consequently, one major treatment aim is the restoration of a body weight within what is defined as the normal range [4]. Depending on the country and treatment setting, different weight gains are recommended ranging between 0.5 and 1.4 kg per week [5].

The refeeding of patients with AN is not only a psychologically but also a metabolically complex process [6]. For instance, during the early phase of refeeding, patients might excessively restore extracellular water (ECW), leading to a rapid increase of body weight [7]. This in turn might result in a massive psychological burden for the patients due to the fear of gaining weight. Moreover, after this initial phase of weight regain, patients with AN might become hyper-metabolic with excessively high energy demands of up to 3000–4000 kcal per day for the restoration of body weight within the normal BMI range [8, 9]. Considering the temporarily rapid weight gain or excessive energy demand as well as the centrality of the fear of weight gain in AN, the entire procedure of refeeding is quite onerous for the patients. Hence, in clinical practice, it is not uncommon to observe weight manipulations such as water loading as a psychopathological symptom of AN before weight measurement.

Bioelectrical impedance analysis (BIA) is an easy-to-use and non-invasive method in order to evaluate body composition and nutritional status [10]. In order to do so, an alternating current is applied to the human body, and the directly measured resistance (R) and reactance (Xc), which together form the total impedance Z, are used to indirectly infer body compartments (e.g. fat mass (FM) or ECW) with suitable formulas that derived from reference samples [11, 12]. Apart from that, the parameters R and Xc can be used directly in their raw form to evaluate nutritional status [10]. The phase angle (PhA), which can be directly derived from R and Xc, represents the quantity and quality of cellular tissue and is the best-established BIA raw parameter [10]. The advantage of these raw parameters is their independence of suitable reference samples and assumptions the formulas for the determination of body compartments are based on [10]. For example, most formulas assume a constant hydration status of the fat-free mass (FFM) of 73% [10, 13]. However, because there has not been any international technological manufacturing-standard (e.g. for the properties of the electrodes) so far, even these raw parameters cannot be compared if they are measured using different devices [10, 14]. Moreover, to obtain valid results, BIA must be performed under standardized conditions. One of these conditions is the performance in a fasting state (including food, drinks and alcohol) for more than eight hours [15].

Because of its economic applicability, BIA is a helpful tool in routinely visualizing changes in body composition during the above-mentioned onerous procedure of refeeding [6]. Moreover, it might carry the potential to routinely detect fluid loading by, for instance, revealing unexpected changes of several BIA parameters. The detection of such patterns could not just alert the treatment team, but could even better be used in the therapeutical process together with the patient in a functional manner. Having an “objective” measure for early detection of such patterns might make it easier to enter this process without straining the relationship between carers and patients, as it is the BIA which just gives “neutral” feedback.

Indeed, the impact of the consumption of fluids or pure water directly before BIA was investigated in several studies [16–29]. However, these studies were quite heterogeneous in terms of the devices used and the type and amount of fluid consumed. Some of these results are even quite contradictory. For instance, the impact of 591 ml of water apparently resulted in an overestimation of fat mass (FM) by 0.5% and no significant increase in Z using foot-to-foot BIA in men [22]. In contrast to these results, the same amount of water led to an overestimation of the percentage of fat mass (%FM) of about 1% as well as Z by 12–15 Ω in men and women of similar age and BMI when a hand-to-foot BIA in standing position was used [24]. In a study by Kutáč [26], the influence of 500 ml of water on BIA outcomes was measured with two types of BIA simultaneously. While there was an increase in FM and %FM and no changes in fat free mass (FFM) and total body water (TBW) measured using a single frequency hand-to-foot BIA in standing position, FM and %FM measurements tended to decrease and FFM and TBW increased using a multi-frequency hand-to-foot BIA in supine position [26]. Thus, results on the impact of fluid consumption before BIA do not seem to be transferable from one BIA device to another one, even if they are applied back-to-back within one measurement protocol.

In our department (LVR-University Clinic of Psychosomatic Medicine, Essen, Germany), in accordance with international guidelines for the treatment of eating disorders, we recommend a body weight increase of 0.5–1 kg/week during inpatient treatment [5]. Moreover, the weekly examination of the body weight gain is routinely monitored in combination with BIA using the medical Body Composition Analyzer (mBCA) 515 (seca GmbH & Co. KG, Hamburg, Germany). As a multi-frequency, eight-electrode segmental scale-type hand-to-foot impedance analyzer, it was validated using gold standard reference methods for the respective body compartments [12, 30]. Yet, and despite its popularity, no systematic investigation has examined the device’s performance in a full experimental design with water intake as a manipulation. For therapeutic and ethical reasons, it is not possible to recruit patients with AN for a technical validation study. We therefore recruited a healthy sample which resembles the AN population as much as possible. Accordingly, young, healthy women within the lower normal BMI range were enrolled in our study. The aim was to examine changes in BIA measurements after drinking 1000 ml of tap water using the seca mBCA 515.

## Materials and methods

### Study participants and recruitment process

Participants were recruited via social media platforms and on the campus of the University of Duisburg-Essen. Interested candidates were screened prior to the study via telephone interview for a set of eligibility criteria. Prospective participants needed to be female, Caucasian, non-pregnant or lactating, 18–25 years old, and have a BMI ranging from 18.5 to 22.0 kg/m^2^. Exclusion criteria were diseases (e.g. diabetes mellitus, kidney failure/renal insufficiency, heart insufficiency or AN), medication intake, which are known to affect the body’s osmoregulation– such as diuretics or corticosteroids– or factors that could influence BIA measurements, including limb amputations, artificial limbs, stents, or electronic implants. Interested candidates were informed about the study procedure, including that they need to arrive at our clinic in a fasting state in the morning on two consecutive days (Saturday and Sunday), be able to drink 1000 ml of water within 15 min, and refrain from alcohol the day before each study day. Furthermore, participants were not allowed to perform any exhausting exercise or visit the sauna the day before each study day to leave osmoregulation in a natural state. Smokers were asked to refrain from consuming nicotine during the experiment.

Altogether, 149 interested candidates were screened, of which 80 fulfilled inclusion criteria during the screening process. Eventually, 64 participated in the study. Sixty-one participants were included in the analysis as the remaining did not fulfill the BMI criteria, did not appear the 2nd study day or reported fluid or food intake after 11 pm the day before.

### Study design and procedure

In the study, we used a full experimental setting with a cross-over design. On both study days, participants completed a self-designed checklist eligibility (e.g. if they remained in a fasting state for at least 8 h).

On both days, all participants underwent a baseline BIA in the fasting state (t_0_). Directly after that, participants were either asked to consume 1000 ml of tap water (intervention condition, “drinking condition”) or to wait for the second BIA measurement (control condition, “waiting condition”) 20 min (t_1_) after baseline measurement. Further BIA measurements took place 40 min (t_2_) and 60 min (t_3_) after the baseline measurement. All participants underwent the treatment and the control procedure on two consecutive days, but the sequence was counterbalanced. While 30 randomly selected participants underwent the treatment condition on day one, the other 31 participants underwent the control procedure on day one. The average temperature of the tap water was 13.9 ± 1.0 (mean ± SD) °C. Weighing of the two drinking bottles (á 500 ml) before and after the water consumption indicated an average net consumption of 998 ± 2 ml. BIA measurement took place 19.9 ± 0.5 min (t_1_), 39.9 ± 0.5 min (t_2_) and 59.6 ± 0.6 min (t_3_) after t_0_, respectively, for the intervention condition, and 19.9 ± 0.6 min (t_1_), 39.9 ± 0.7 min (t_2_) and 59.5 ± 0.6 min (t_3_) after t_0_, respectively, for the control condition. Measurement was performed barefoot in light clothing, and participants were asked to wear the same clothes on both days. Moreover, participants were asked to be seated for 15 min before BIA measurement.

### Bioelectrical impedance analysis (BIA) & anthropometrics

The seca mBCA 515 (seca GmbH & Co. KG, Hamburg, Germany) was used for BIA measurements. This device is designed for measurement in a standing position. It uses an eight-electrode technique with each two electrodes on both sides at the handle and the platform enabling segmental impedance measurements of the torso, arms and legs with a current of 100 µA and frequencies of 5 and 50 kHz. The duration of each measurement is 17 s. Body mass is rounded to the nearest 50 g.

Body compartments were received by proprietary equations of seca, which were established using gold standard reference methods for the respective body compartments among Caucasians adults with a BMI range of 18.5–35 kg/m^2^ [12, 30]. Data were extracted using seca’s proprietary software (seca analytics 115 version 1.4.1040.6789, seca GmbH & Co. KG, Hamburg, Germany). Outcome measures were body mass, FM, FFM, skeletal muscle mass (SMM), TBW, extracellular water (ECW), ECW/TBW, and PhA.

Height was measured once at the beginning of the study using a stadiometer (seca GmbH & Co. KG, Hamburg, Germany). Waist circumference (WC) was obtained midway between the lowest rib and the uppermost boarder of the iliac crest using a non-stretchable tape (circumference measuring tape seca 201, seca GmbH & Co. KG, Hamburg, Germany) at each BIA measurement. Both height and WC were rounded to nearest cm.

Visceral adipose tissue (VAT) can also be estimated with seca’s mBCA 515 [30]. WC is generally considered a good estimate of VAT [37]. Hence, as we found a low and even negative correlation between VAT and WC among our study participants at baseline, VAT was not considered for further analyses.

### Statistics

Statistical analyses were performed using R version 4.4.1. In a first step, we tested for potential carryover effects using Grizzle’s method at t_1_, t_2_ and t_3_, i.e. calculating the sum of each parameter on the first and second measurement day and comparing the sequence groups (control > treatment vs. treatment > control) against each other using a t-test [31]. Despite the alpha-error accumulation, all the tests yielded p-values– mostly far– beyond a threshold of α = 0.1 / 0.15 indicating no carryover effect. We reproduced this finding using mixed linear models with time of measurement, condition and period, as well as generalized estimating equations with the t_0_-measurement as a covariate, a carryover effect specifying the explicit effect of water consumption on measurement day one on measurement day two, as well as the period effect, as proposed in the R package CrossCarry [32]. Again, we did not find any indications that carryover effects might exist. Accordingly, observations from both study days were considered in our main analysis. This main analysis on the intervention effect was conducted using repeated-measures ANOVA models including time (t_0_ vs. t_1_ vs. t_2_ vs. t_3_), condition (treatment vs. control) as well as their interaction with Greenhouse-Geisser corrections for non-sphericity. To subsequently disentangle interaction effects, simple effect analyses and planned contrasts were performed. Here, Sidak-corrections were applied for planned comparisons within a condition for each measurement point against t_0_ (= “within-condition comparison”), as well as between the conditions, i.e. between intervention and control condition for each measurement point (= “between-condition comparison”). Corrections for multiple testing were not performed due to the exploratory character of this study. In the following, we will always report the mean ± SD.

### Ethical considerations

The study was approved by the Ethics Committee of the medical faculty of the University of Duisburg-Essen (approval numbers 20-9525-BO, 20-9525_1-BO and 20-9525_2-BO). All participants gave written informed consent to the study conditions before participating in the study.

## Results

Study participants (*n* = 61) had an average age of 22.2 ± 2.2 years and BMI of 20.4 ± 1.0 kg/m^2^, respectively, at baseline on the first of the two consecutive study days. Values of all BIA parameters can be found in the appendix (Table S1). Coefficients of variation of the two baseline values each ranged between 0.4 and 1.8% (1.1 ± 0.5%). Intra-day coefficients of variation of the four measurements ranged between 0.5 and 3.1% (1.1 ± 0.8%) for the drinking condition and 0.1–1.8% (0.8 ± 0.5%) for the waiting condition.

Repeated-measures ANOVAs on the intervention effect yielded significant time effects in all eight outcome parameters (body mass, FM, FFM, SMM, TBW, ECW, ECW/TBW, and PhA) and significant main effects for condition variable in five of them (body mass, FM, FFM, TBW, and ECW). Moreover, significant time x condition interaction effects were found in all parameters except for ECW/TBW (Table S2).

**Fig. 1:**
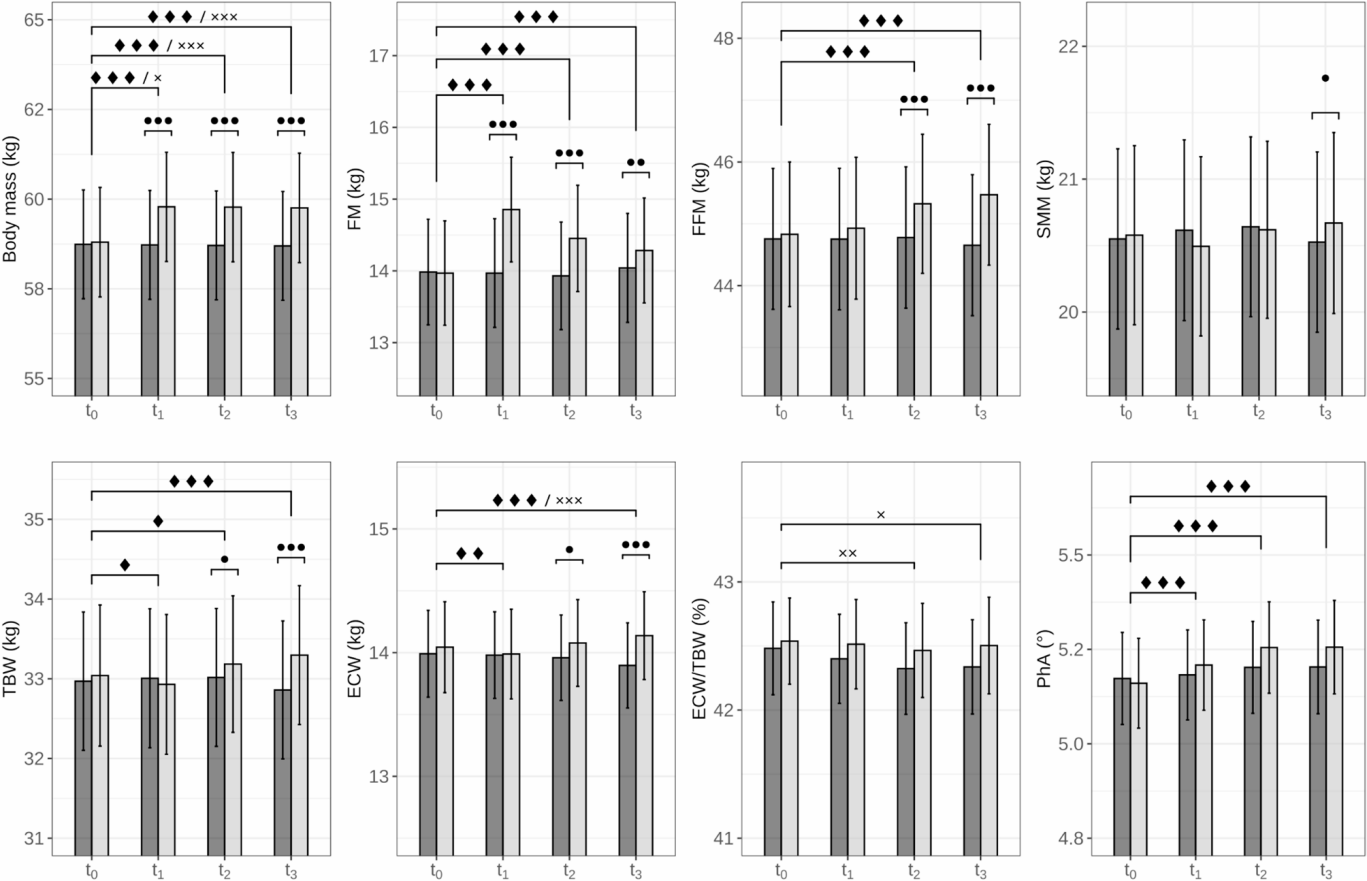
Course of all outcome parameters for control (dark grey) and intervention condition (light grey). ♦, ♦♦, ♦♦♦ indicate significant differences compared to baseline for intervention condition at *p* < 0.5, 0.01, 0.001, respectively, and **x**, **x x**, **x x x**, significant differences compared to baseline for control condition at *p* < 0.5, 0.01, 0.001, respectively, determined by Sidak-corrections. •, ••, ••• indicate significant differences between intervention and control condition at specific time point at *p* < 0.5, 0.01, 0.001, respectively, determined by Sidak-corrections. ECW, extracellular water; FFM, fat free mass; FM, fat mass; PhA, phase angle; SMM, skeletal muscle mass; TBW, total body water

In more detail, the consumption of 1000 ml of water before BIA resulted in an average increase of body mass by each 0.98 ± 0.04 kg at t_1_ and t_2_, and by 0.95 ± 0.04 kg at t_3_ compared with t_0_, respectively. In the control condition, body mass decreased by 0.02–0.04 kg. Changes from t_0_ were significant for both conditions at each measurement point (Table 1; Fig. 1). Between-condition comparisons suggested significant differences between the drinking and waiting condition during t_1_, t_2_ and t_3_, but not at t_0_ (Fig. 1).

A similar pattern was found for FM measurements with average increases by 0.89 ± 0.34 kg, 0.48 ± 0.43 kg, and 0.31 ± 0.44 kg at t_1_, t_2_, and t_3_, respectively, during the drinking condition. By contrast, FM had a comparably stable course during the waiting condition. Within-condition comparisons against t_0_ were only significant in the drinking condition (Table 1; Fig. 1). Between-condition differences occurred at all measurement points except for t_0_ (Fig. 1).

FFM, by contrast, increased continuously from t_1_ to t_3_ during the drinking condition with significant increases against t_0_ only at t_2_ and t_3_, while it was stable during the waiting condition (Table 1; Fig. 1). Between-condition differences also only occurred at t_2_ and t_3_ (Fig. 1).

The course of SMM showed a relatively constant pattern for both conditions. Equally, within-condition differences were not found reflecting the relatively stable patterns during both conditions (Table 1; Fig. 1). Between-condition differences only occurred at t_3_ (Fig. 1).

Concerning different body water measurements, TBW and ECW exhibited similar patterns during the drinking condition with slight decreases below the respective baseline value at t_1_, and continuous increases above the respective baseline values at t_2_ and t_3_. In turn, within-condition comparisons against t_0_ showed significant differences for TBW at t_1_, t_2_, and t_3_ and for ECW at t_1_ and t_3_ during the drinking condition. During the waiting condition, values remained rather constant except for a significant decrease of ECW at t_3_ by -0.09 ± 0.16 kg (Table 1; Fig. 1). Between-condition differences only occurred at t_2_ and t_3_ for both TBW and ECW (Fig. 1). The ratio of ECW by TBW (ECW/TBW) had a comparably stable pattern during the drinking condition, while values were significantly decreased against t_0_ at t_2_ and t_3_ during the waiting condition (Table 1; Fig. 1). Between-condition comparisons at each time point remained statistically insignificant (Fig. 1).

Regarding the raw parameters, PhA values increased continuously by up to 0.10 ± 0.11° during the drinking condition and remained rather stable during the waiting condition. Within-condition comparisons against t_0_ exhibit a continuous increase in the drinking condition, but no effects during the waiting condition (Table 1; Fig. 1). Between-condition differences were not found (Fig. 1). Similarly, all remaining raw parameters, Z, R and Xc, had a comparable pattern with continuous and significant increases during the drinking condition and relatively stable courses during the waiting condition (Fig. S1).

Taken together, all eight outcome parameters deviated from the baseline value at t_1_, t_2_, and t_3_ by 1.3 ± 2.2%, 1.2 ± 1.2% and 1.2 ± 0.8%, respectively, during the drinking condition, and only by 0.1 ± 0.1%, 0.3 ± 0.2% and 0.3 ± 0.2%, respectively, during the waiting condition. During the drinking condition, relative changes of FM were highest at each measurement point (2.4 to 6.6%), while those of ECW/TBW were lowest (-0.1 to -0.2%). During the waiting condition, the most pronounced change was that of ECW by -0.7% at t_3_ (Table 2).

**Table 1:** Within-condition differences compared to baseline values (absolute values)

	Intervention			Control		
	Δt_1_-t_0_	Δt_2_-t_0_	Δt_3_-t_0_	Δt_1_-t_0_	Δt_2_-t_0_	Δt_3_-t_0_
**Body mass (kg)**	0.98 ± 0.04***	0.98 ± 0.04***	0.95 ± 0.04***	-0.02 ± 0.05*	-0.03 ± 0.04***	-0.04 ± 0.04***
**FM (kg)**	0.89 ± 0.34***	0.48 ± 0.43***	0.31 ± 0.44***	-0.01 ± 0.41	-0.05 ± 0.40	0.06 ± 0.42
**FFM (kg)**	0.10 ± 0.34	0.49 ± 0.44***	0.64 ± 0.45***	0.00±0.41	0.02 ± 0.39	-0.10 ± 0.41
**SMM (kg)**	-0.08 ± 0.25	0.04 ± 0.35	0.09 ± 0.38	0.07 ± 0.35	0.09 ± 0.32	-0.02 ± 0.33
**TBW (kg)**	-0.11 ± 0.30*	0.14 ± 0.40*	0.26 ± 0.42***	0.04 ± 0.40	0.05 ± 0.36	-0.11 ± 0.38
**ECW (kg)**	-0.06 ± 0.11**	0.03 ± 0.14	0.09 ± 0.14***	-0.01 ± 0.14	-0.03 ± 0.15	-0.09 ± 0.16***
**ECW/TBW (%)**	-0.02 ± 0.26	-0.07 ± 0.31	-0.03 ± 0.41	-0.08 ± 0.33	-0.16 ± 0.37**	-0.14 ± 0.36*
**PhA (°)**	0.05 ± 0.07***	0.09 ± 0.08***	0.10 ± 0.11***	0.01 ± 0.10	0.03 ± 0.11	0.03 ± 0.11

ECW, extracellular water; FFM, fat free mass; FM, fat mass; PhA, phase angle; SMM, skeletal muscle mass; TBW, total body water; * *p* < 0.05, ** *p* < 0.01, *** *p* < 0.001 determined by Sidak-corrections for planned comparisons between conditions for each measurement point against t_0_

**Table 2:** Within-condition differences compared to baseline values (in %)

	Intervention			Control		
	Δt_1_-t_0_	Δt_2_-t_0_	Δt_3_-t_0_	Δt_1_-t_0_	Δt_2_-t_0_	Δt_3_-t_0_
**Body mass (kg)**	1.7 ± 0.2	1.7 ± 0.2	1.6 ± 0.2	0.0 ± 0.1	-0.1 ± 0.1	-0.1 ± 0.1
**FM (kg)**	6.6 ± 3.0	3.6 ± 3.5	2.4 ± 3.8	-0.2 ± 3.2	-0.4 ± 3.0	0.3 ± 3.2
**FFM (kg)**	0.2 ± 0.8	1.2 ± 1.0	1.5 ± 1.1	0.0 ± 0.9	0.1 ± 0.9	-0.2 ± 0.9
**SMM (kg)**	-0.4 ± 1.3	0.2 ± 1.7	0.4 ± 1.9	0.3 ± 1.8	0.5 ± 1.6	-0.1 ± 1.7
**TBW (kg)**	-0.3 ± 0.9	0.5 ± 1.2	0.8 ± 1.3	0.1 ± 1.3	0.2 ± 1.1	-0.3 ± 1.2
**ECW (kg)**	-0.4 ± 0.8	0.3 ± 1.0	0.7 ± 1.0	-0.1 ± 1.0	-0.2 ± 1.1	-0.7 ± 1.1
**ECW/TBW (%)**	-0.1 ± 0.6	-0.2 ± 0.7	-0.1 ± 0.9	-0.2 ± 0.8	-0.4 ± 0.9	-0.3 ± 0.9
**PhA (°)**	0.9 ± 1.4	1.8 ± 1.6	1.9 ± 2.1	0.2 ± 1.8	0.6 ± 2.2	0.6 ± 2.3

ECW, extracellular water; FFM, fat free mass; FM, fat mass; PhA, phase angle; SMM, skeletal muscle mass; TBW, total body water

## Discussion

Water loading before weight measurement is a common “strategy” of patients with AN in a context where weight gain is expected. To overcome fear of weight gain and allow for more precise measurement, a visualization of such (un)conscious manipulation might be helpful in the therapeutic process of the patient. BIA is not only an economic tool in routinely visualizing changes in body composition during the refeeding process but could also potentially detect water loading. With our study, we aimed at investigating the influence of the consumption of 1000 ml of water on BIA outcomes among young, healthy and normal weight women in an experimental setting. We found an immediate increase of measured FM and PhA, and a delayed increase of FFM, ECW and TBW, while SMM as well as ECW/TBW remained rather stable.

Several studies were performed on the effect of fluid or pure water consumption before BIA, but results varied depending of the type of BIA used, i.e. leg-to-leg (foot-to-foot) [22] vs. hand-to-foot BIA [24] or hand-to-foot BIA in standing vs. supine position [26]. Therefore, we will focus our comparison on BIA devices using a similar procedure as that of the seca mBCA 515 used in our study– i.e. a scale-type hand-to-foot contact electrode BIA in standing position [24, 26].

Regarding FM, our results are in line with the findings of two previous studies [24, 26]. The immediate increase of measured FM of 0.89 kg post consumption of 1000 ml of water in our study had a similar magnitude compared with the increase of 0.4 kg 10–12 min after consumption of water of 500 ml reported by Kutáč [26]. According to Tedner and Lins [33], fluid intake was only gradually visible after 15–30 min using an impedance fluid monitor. The authors stated that consumed fluids cannot be measured as fluids/body water as long as they reside in the stomach or gastrointestinal tract and bladder, as only metabolically active tissue is captured by impedance measurement [33]. In most BIA equations, FM is calculated as difference between body mass and FFM [34]. Thus, the artificial increase of FM, which was most pronounced 20 min after baseline measurement in our study, might be the result of water still residing in the stomach or gastrointestinal tract and therefore adding to the body weight but not to FFM.

Moreover, this assumption might also explain why the overestimation of FM gradually decreases after peaking at 20 min, and FFM and TBW (as a major compartment of FFM) only increased 40 min and 60 min after baseline measurement in our study. In a similar fashion, FFM and TBW remained unchanged 10–12 min after baseline measurement in the study of Kutáč [26]. Interestingly, Dixon and colleagues found a continuous decrease of TBW by 0.2–0.4 kg after the consumption of water 20–60 min after baseline measurement [24]. These surprisingly contradictory outcomes might be the result of several differences between this study and ours, such as different BIA devices with proprietary equations to infer body compartments, different study protocols pertaining to participants (men and women [24] vs. only women in our study) or different amounts of consumed water (591 ml [24] vs. 1000 ml in our study).

The remaining BIA parameters considered in our study (ECW, ECW/TBW, SMM, and PhA) were not assessed in the two other studies using hand-to-foot BIA in standing position [24, 26]. Therefore, we are not able to evaluate these by direct comparison. Regarding the BIA raw parameters, we found immediate increases in Z as well as all other raw parameters including PhA after water consumption, which remained elevated until 60 min after baseline measurement (Fig. S1). Similarly, Dixon and colleagues found Z to be elevated after water consumption up to 60 min after baseline measurement [24]. Using hand-to-foot BIA in a supine position, Kutáč reported body cell mass (BCM), and extracellular mass (ECM) divided by BCM, which corresponds to SMM, and ECW/TBW, respectively, in our study. However, as previously noted, the simultaneous measurements taken within one study protocol produced varying outcomes, depending on whether BIA was performed in standing or supine position [26]. Therefore, comparing results from different protocols may not yield reliable conclusions. The contradictory results concerning TBW as well as the fact that previous studies did not report all BIA parameters used in our clinical routine underpinned the necessity to perform such a study with a BIA device used in our clinical routine.

The natural course of all outcome parameters could be detected in the control condition of our study. Significant changes were noted for three of the eight outcome parameters, namely a decrease of ECW 60 min as well as ECW/TBW 40 min and 60 min after baseline measurement and body mass at each measurement point after baseline measurement. The estimated decrease of ECW of 90 g might have resulted from a loss of body water via lung, skin or sweat and hence a process with a natural cause. Moreover, this decrease might have been responsible for the decreased ECW/TBW as well as the loss of body mass of up to 40 g during the one-hour study period of our study.

Although we used a full experimental setting for our study, we aimed at imitating the clinical routine regarding the weekly weight monitoring process of the patients with AN in our department as much as possible. For this reason, we performed the study in the morning, when weighing is normally performed, using the same BIA device and tap water, which is calorie-free and available at any time and hence, the most likely means for water loading for the patients in our department. Nevertheless, the results of our study might not be one-to-one transferable to the clinical setting for several reasons. First, generalizability to patients with AN might be limited, as these patients could show distinct physiological patterns after the consumption of water compared to the normal weight, young women included in our study. Lower BMI values, a potentially different body composition and varying metabolism– also depending on the phase of refeeding– might lead to different outcomes among patients with AN.

In addition, we are unable to comment on the relationship between the amount of water consumed and the change in parameters, as all participants consumed the same amount of water. Future research should address this relationship.

Moreover, as osmolarity has an influence on stomach emptying and absorption of fluids [35], our results might not be transferable to fluids other than tap water as for instance rehydration solutions containing carbohydrates with or without sodium. Nevertheless, as mentioned above, being free of calories and at any time available for the patients in our department, tap water might be the most favorite choice for fluid loading among patients with AN.

Finally, it remains unclear to which extent a BIA measurement could distinguish between a true weight gain of 1 kg and a weight gain due to water loading. According to Mika and colleagues [36], adolescent patients with AN with an average weight gain of approximately 5.2 kg, had an increase of FM and BCM of 4.1 kg and 1.7 kg, respectively, within 12 weeks of refeeding. Transferring to our data, both an increase of FM and SMM should be expected by a true weight gain of 1 kg. However, the extent of change of each body compartment might differ individually and week by week. Therefore, our findings warrant further research to determine whether the significant increase in FM accompanied by relatively stable SMM, observed in our study following water consumption, could potentially indicate water loading.

## Conclusion

In conclusion, our study provided insight into the changes of impedance raw data and derived body compartments after the consumption of 1000 ml of water among healthy, young and normal weight women. Our results form a basis for the implementation of detecting water loading in patients with AN using BIA. However, although the considerable increase of FM in combination with a rather constant course of SMM could be a potential hint for water loading, the outcomes of our experiment are not yet one-to-one transferable to the clinical setting, and further investigation to what extent these results are applicable in patients with AN is necessary.

## Electronic supplementary material

Below is the link to the electronic supplementary material.

Supplementary Material 1

## Data Availability

Raw data and R Syntax are available from the corresponding author on reasonable request.

## Declarations

### Trial registration

The study is registered at the German Clinical Trials Register (DRKS-ID: DRKS00030472). Date of registration: October 10th, 2022, retrospectively registered.

## Abbreviations

AN: Anorexia nervosa
BIA: Bioelectrical impedance analysis
BMI: Body mass index
ECW: Extracellular water
FFM: Fat free mass
FM: Fat mass
PhA: Phase angle
R: Resistance
SMM: Skeletal muscle mass
TBW: Total body water
VAT: Visceral adipose tissue
WC: Waist circumference
Xc: Reactance
Z: Impedance
