# Supplementary material for: Can bioelectrical impedance analysis be used to identify water loading in patients with anorexia nervosa?– Implications from experimental measurements in young, healthy, and normal weight women
Source: J Eat Disord. 2025 May 19;13:87. doi: 10.1186/s40337-025-01285-z (PMC12090468; doi:10.1186/s40337-025-01285-z)
Supplement: Supplementary file 1 — Supplementary Material 1 [file 40337_2025_1285_MOESM1_ESM.docx]

**Supplements**

**Table S1 Average values of all outcome parameters and weight circumference during the intervention and control**

|  | Intervention | | | | Control | | | |
| --- | --- | --- | --- | --- | --- | --- | --- | --- |
| N=61 | t_0_ | t_1_ | t_2_ | t_3_ | t_0_ | t_1_ | t_2_ | t_3_ |
| **Body mass (kg)** | 58.80 (5.96) | 59.78 (5.95)*** | 59.78 (5.95)*** | 59.75 (5.96)*** | 58.74 (5.92) | 58.72 (5.93)* | 58.71 (5.92)*** | 58.70 (5.92)*** |
| **FM (kg)** | 13.97 (2.84) | 14.85 (2.85)*** | 14.45 (2.89)*** | 14.28 (2.85)*** | 13.98 (2.87) | 13.97 (2.96) | 13.93 (2.93) | 14.04 (2.97) |
| **FFM (kg)** | 44.83 (4.57) | 44.93 (4.48) | 45.32 (4.39)*** | 45.47 (4.45)*** | 44.76 (4.45) | 44.75 (4.47) | 44.78 (4.46) | 44.65 (4.45) |
| **SMM (kg)** | 20.58 (2.63) | 20.49 (2.63) | 20.62 (2.60) | 20.67 (2.66) | 20.55 (2.65) | 20.62 (2.65) | 20.64 (2.64) | 20.53 (2.65) |
| **TBW (kg)** | 33.04 (3.46) | 32.93 (3.42)* | 33.18 (3.34)* | 33.30 (3.40)*** | 32.97 (3.39) | 33.01 (3.40) | 33.02 (3.38) | 32.86 (3.38) |
| **ECW (kg)** | 14.04 (1.43) | 13.99 (1.42)** | 14.08 (1.37) | 14.14 (1.39)*** | 13.99 (1.37) | 13.98 (1.37) | 13.96 (1.35) | 13.90 (1.34)*** |
| **ECW/TBW (%)** | 42.54 (1.31) | 42.51 (1.36) | 42.46 (1.43) | 42.50 (1.47) | 42.48 (1.42) | 42.40 (1.36) | 42.32 (1.40)** | 42.34 (1.44)* |
| **PhA (°)** | 5.16 (0.46) | 5.21 (0.47)*** | 5.25 (0.47)*** | 5.26 (0.48)*** | 5.17 (0.48) | 5.18 (0.47) | 5.20 (0.48) | 5.20 (0.48) |

ECW, extracellular water; FFM, fat free mass; FM, fat mass; PhA, phase angle; SMM, skeletal muscle mass; TBW, total body water; WC, weight circumference; Values expressed as mean (SD);

* p<0.05, ** p<0.01, *** p<0.001 determined by Sidak-corrections for planned comparisons between conditions for each measurement point against t_0_

**Table S2 Time, condition and interaction effects of all outcome parameters**

| **Outcome parameter** | **Effect** | **Df** | **MSE** | **F** | **η^2^** | **p.value** |
| --- | --- | --- | --- | --- | --- | --- |
| **Body mass (kg)** | time | 2.79, 167.27 | 0.00 | 7353.55 *** | .001 | <.001 |
|  | condition | 1, 60 | 0.44 | 183.30 *** | .005 | <.001 |
|  | time:condition | 2.80, 168.25 | 0.00 | 8847.42 *** | .001 | <.001 |
| **FM (kg)** | time | 2.29, 137.51 | 0.11 | 46.27 *** | .003 | <.001 |
|  | condition | 1, 60 | 0.34 | 60.36 *** | .005 | <.001 |
|  | time:condition | 2.60, 156.00 | 0.07 | 71.89 *** | .003 | <.001 |
| **FFM (kg)** | time | 2.26, 135.81 | 0.11 | 28.66 *** | <.001 | <.001 |
|  | condition | 1, 60 | 0.51 | 38.89 *** | .002 | <.001 |
|  | time:condition | 2.59, 155.28 | 0.07 | 58.90 *** | .001 | <.001 |
| **SMM (kg)** | time | 2.42, 144.94 | 0.06 | 3.00 * | <.001 | .043 |
|  | condition | 1, 60 | 0.23 | 0.03 | <.001 | .874 |
|  | time:condition | 2.60, 156.29 | 0.05 | 8.43 *** | <.001 | <.001 |
| **ECW (kg)** | time | 2.24, 134.16 | 0.01 | 3.62 * | <.001 | .025 |
|  | condition | 1, 60 | 0.22 | 6.21 * | .001 | .016 |
|  | time:condition | 2.50, 149.74 | 0.01 | 49.23 *** | .001 | <.001 |
| **TBW (kg)** | time | 2.42, 145.37 | 0.08 | 7.17 *** | <.001 | <.001 |
|  | condition | 1, 60 | 0.33 | 8.24 ** | <.001 | .006 |
|  | time:condition | 2.55, 153.02 | 0.06 | 26.37 *** | <.001 | <.001 |
| **ECW/TBW (%)** | time | 2.13, 128.05 | 0.08 | 5.58 ** | <.001 | .004 |
|  | condition | 1, 60 | 1.08 | 1.61 | .002 | .209 |
|  | time:condition | 2.55, 153.21 | 0.05 | 1.37 | <.001 | .255 |
| **PhA (°)** | time | 1.78, 107.07 | 0.01 | 23.03 *** | .003 | <.001 |
|  | condition | 1, 60 | 0.06 | 1.81 | <.001 | .183 |
|  | time:condition | 2.69, 161.10 | 0.00 | 9.76 *** | <.001 | <.001 |

ECW, extracellular water; FFM, fat free mass; FM, fat mass; PhA, phase angle; SMM, skeletal muscle mass; TBW, total body water; * p<0.05, ** p<0.01, *** p<0.001


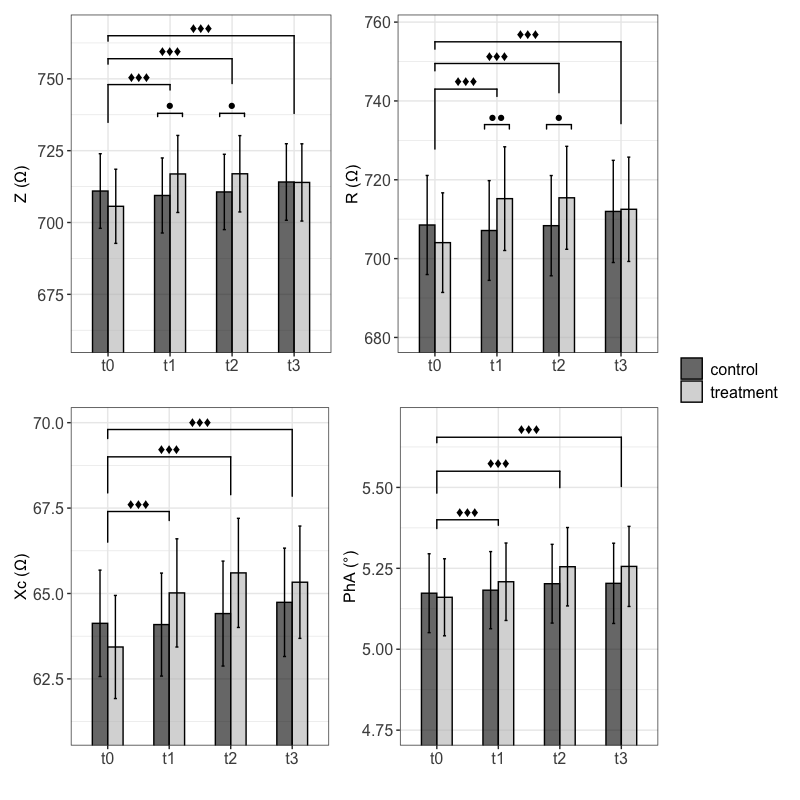


**Fig. S1** **Course of Z, R, Xc, and PhA for control (dark grey) and intervention condition (light grey)**

♦, ♦♦, ♦♦♦ indicate significant difference from baseline (intervention condition) at p < 0.5, 0.01, 0.001, respectively, determined by Sidak-corrections

•, ••, •••, indicate significant difference between intervention and control condition at specific time point at p < 0.5, 0.01, 0.001, respectively, determined by Sidak-corrections

Xc, reactance; R, resistance; Z, total impedance, PhA, phase angle
